# Dual-Specificity, Tyrosine Phosphorylation-Regulated Kinases (DYRKs) and cdc2-Like Kinases (CLKs) in Human Disease, an Overview

**DOI:** 10.3390/ijms22116047

**Published:** 2021-06-03

**Authors:** Mattias F. Lindberg, Laurent Meijer

**Affiliations:** Perha Pharmaceuticals, Perharidy Peninsula, 29680 Roscoff, France; lindberg@perha-pharma.com

**Keywords:** DYRKs, CLKs, kinase, kinase inhibitor, Alzheimer’s disease, Down syndrome, type 1 diabetes, type 2 diabetes, acute lymphoblastic leukemia, viral infections

## Abstract

Dual-specificity tyrosine phosphorylation-regulated kinases (DYRK1A, 1B, 2-4) and cdc2-like kinases (CLK1-4) belong to the CMGC group of serine/threonine kinases. These protein kinases are involved in multiple cellular functions, including intracellular signaling, mRNA splicing, chromatin transcription, DNA damage repair, cell survival, cell cycle control, differentiation, homocysteine/methionine/folate regulation, body temperature regulation, endocytosis, neuronal development, synaptic plasticity, etc. Abnormal expression and/or activity of some of these kinases, DYRK1A in particular, is seen in many human nervous system diseases, such as cognitive deficits associated with Down syndrome, Alzheimer’s disease and related diseases, tauopathies, dementia, Pick’s disease, Parkinson’s disease and other neurodegenerative diseases, Phelan-McDermid syndrome, autism, and CDKL5 deficiency disorder. DYRKs and CLKs are also involved in diabetes, abnormal folate/methionine metabolism, osteoarthritis, several solid cancers (glioblastoma, breast, and pancreatic cancers) and leukemias (acute lymphoblastic leukemia, acute megakaryoblastic leukemia), viral infections (influenza, HIV-1, HCMV, HCV, CMV, HPV), as well as infections caused by unicellular parasites (*Leishmania*, *Trypanosoma*, *Plasmodium*). This variety of pathological implications calls for (1) a better understanding of the regulations and substrates of DYRKs and CLKs and (2) the development of potent and selective inhibitors of these kinases and their evaluation as therapeutic drugs. This article briefly reviews the current knowledge about DYRK/CLK kinases and their implications in human disease.

## 1. Introduction

### 1.1. Protein Phosphorylation, Protein Kinases, Kinase Inhibitors, and Human Disease

**Figure 1:**
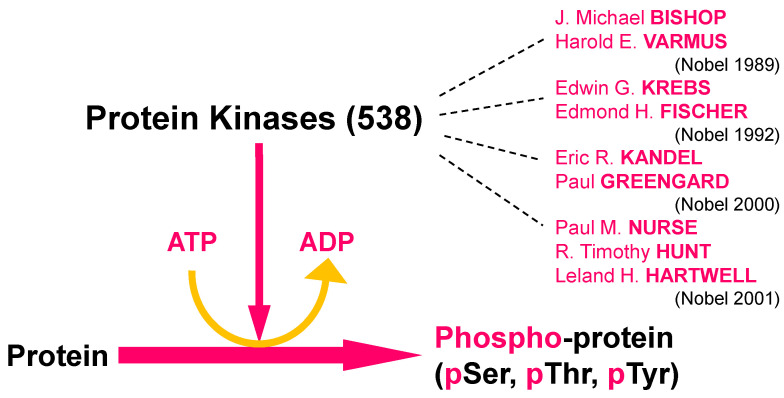
Four Nobel Prizes in Physiology or Medicine awarded in the field of protein phosphorylation and protein kinases. Protein kinases catalyze the transfer of the γ-phosphate of ATP to the hydroxyl substituents of serine, threonine, or tyrosine residues in proteins, thereby altering the physiological properties of their protein substrates. The human kinome comprises 538 protein kinases. Michael Bishop and Harold E. Varmus received the Nobel Prize 1989 “for their discovery of the cellular origin of retroviral oncogenes” (src, the first described oncogene, which encodes a tyrosine kinase). Edmond H. Fischer and Edwin G. Krebs received the Nobel Prize 1992 “for their discoveries concerning reversible protein phosphorylation as a biological regulatory mechanism” (they are the true discoverers of protein kinases). The Nobel Prize 2000 was awarded jointly to Arvid Carlsson, Paul Greengard, and Eric R. Kandel “for their discoveries concerning signal transduction in the nervous system” (Paul Greengard investigated the mechanism of signal transduction of neurotransmitters in the central nervous system and demonstrated the key importance of phosphorylation by kinases such as CDK5, PKA, CK1, and CK2 and Eric Kandel the importance of PKA in memory in *Aplysia*). The Nobel Prize 2001 was awarded jointly to Leland H. Hartwell, Tim Hunt, and Paul M. Nurse “for their discoveries of key regulators of the cell cycle” (using yeast or sea urchin embryos, they discovered how the cell division cycle is regulated by CDKs). For more information on each of these awardees, see: https://www.nobelprize.org/prizes/medicine/ (accessed on 1 June 2021).

Protein phosphorylation is probably one of the most important and most studied mechanism used by cells to regulate their proteins in terms of enzymatic activity, functions, localization, half-life, interactions with other proteins or other ligands, etc. It is also a key mechanism for signal transduction between cells and within cells. Protein phosphorylation occupies a central place in the scientific literature with 337,916 references (as of 1 June 2021). Protein phosphorylation on serine, threonine, and tyrosine residues is carried out by protein kinases, a family of enzymes known as the human kinome, comprising at least 538 members [1,2] divided into tyrosine kinases and serine/threonine kinases (some of the latter are so-called dual specificity, as they also phosphorylate tyrosine residues), histidine kinases, and pseudo-kinases (protein kinases: 573,472 references (as of 1 June 2021), i.e., one article published every 7 min for the last five years). Quite uniquely, four different Nobel Prizes in medicine or physiology have been awarded to this field (1989, 1992, 2000, 2001) (Figure 1).

Since protein phosphorylation is involved in essentially all physiological events, abnormal phosphorylation is implicated in many human diseases. Abnormally expressed or abnormally active kinases represent the most frequent situation. Consequently, inhibiting disease-relevant kinases or normalizing their activities constitutes a rational approach to tackle numerous diseases. This is why protein kinases have become, in a few decades after their initial discovery [3], the first therapeutic targets—before G-protein-coupled receptors–in the pharmaceutical industry’s search for novel drug candidates (reviews: [2,4,5,6,7,8]). As of early February 2021, 62 kinase inhibitors have reached the market, mostly for the treatment of various cancer indications [9,10,11].

### 1.2. DYRKs and CLKs: Structure, Activation, Interactors, and Substrates

**Figure 2:**
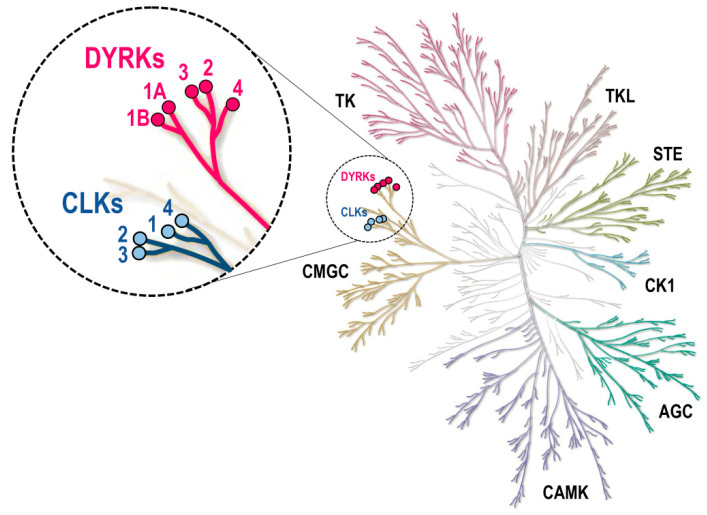
DYRKs and CLKs within the human kinome phylogenetic tree. DYRK and CLK family members are highlighted with pink and blue circles, respectively. Kinome tree: courtesy of Cell Signaling Technology, Inc. (Danvers, MA, USA, www.cellsignal.com, accessed on 1 June 2021). AGC, cAMP-dependent protein kinase (PKA), cGMP-dependent protein kinase (PKG), and protein kinase C (PKC) families; CAMK, Ca^2+^/calmodulin-dependent kinases; CK1, casein kinases 1; CMGC, cyclin-dependent kinases (CDKs), mitogen-activated protein kinases (MAPK), glycogen synthase kinases (GSK3), dual-specificity, tyrosine phosphorylation-regulated kinases (DYRKs) and Cdc2-like kinases (CLKs); STE, homologs of yeast STE20 (MAP4K), STE11 (MAP3K), and STE7 (MAP2K) kinases; TK, tyrosine kinases; TKL, tyrosine kinase-like kinases.

**Figure 3:**
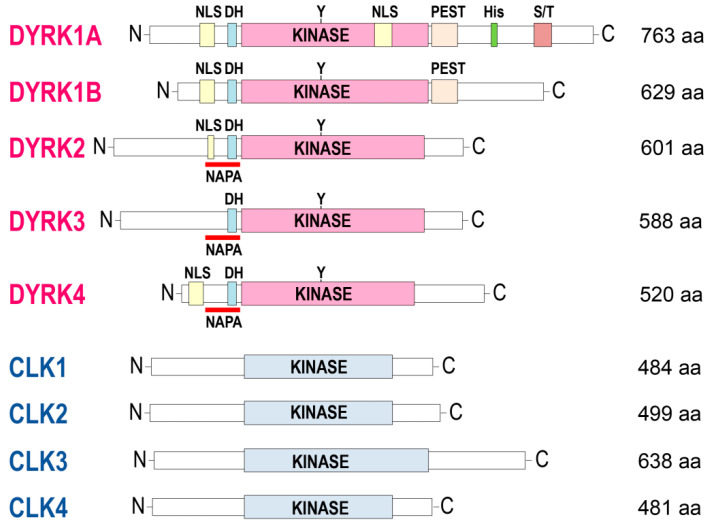
Comparison of DYRKs and CLKs overall structures. Schematic representation of the canonical protein sequences of human CLKs and DYRKs (extracted from uniprot.org). NB: a NLS in DYRK4 is only found in isoform 4, the canonical sequence being isoform 5. aa, amino acids; C, C-terminal; DH, DYRK homology box; His, His domain (13 consecutive histidine residues region); KINASE, kinase domain; N, N-terminal; NAPA, N-terminal autophosphorylation accessory domain; NLS, nuclear localization signals domain; PEST, proline (P), glutamic acid (E), serine (S), and threonine (T)-rich domain; S/T, serine, and threonine-enriched domain; Y, Tyrosine residue autophosphorylated by DYRKs within the activation loop.

**Figure 4:**
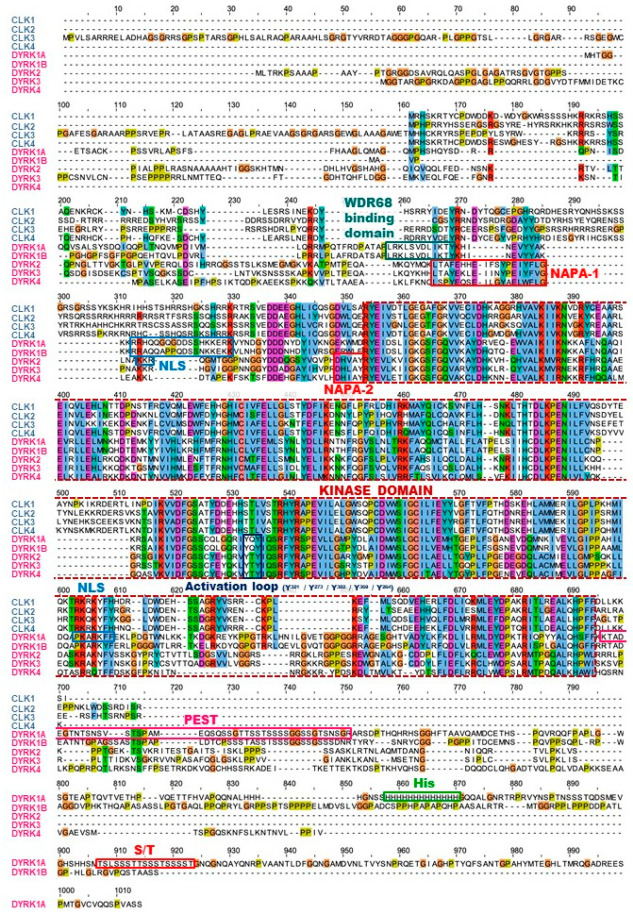
Sequence alignment of human DYRKs and CLKs. Multiple sequence alignment of the canonical sequences of DYRK and CLK members was performed using Clustal Omega [41] (https://www.ebi.ac.uk) (accessed on 12 April 2021) and edited using Jalview [42]. Each residue in the alignment is assigned a colour if the amino acid profile of the alignment at that position meets some minimum criteria specific for the residue type (Clustal X Colour Scheme, http://www.jalview.org/help/html/colourSchemes/clustal.html) (accessed on 12 April 2021). Distinct sequences are indicated: Activation loop and tyrosine residue that is autophosphorylated (Yn); DH, DYRK homology box; His domain, 13 consecutive histidine residues region; kinase domain; NAPA, N-terminal autophosphorylation accessory domain; NLS, nuclear localization signal domain (NB: a NLS sequence is only found in isoform 4 of DYRK4, not in the canonical sequence); PEST, proline (P), glutamic acid (E), serine (S), and threonine (T)-rich domain; S/T, serine, and threonine-enriched domain; WDR68 binding domain.

Among serine/threonine kinases, DYRKs and CLKs (Figure 2, Figure 3 and Figure 4) belong to a family of 62 kinases known as the CMGC group, which also includes mitogen-activated protein kinases (MAPKs), cyclin-dependent kinases (CDKs), and the glycogen synthase kinases 3 (GSK3) family. DYRKs and CLKs are two highly related and conserved kinase families (Table 1), usually sensitive to the same pharmacological inhibitors. The DYRK family comprises 5 members: DYRK1A and DYRK1B (class 1 DYRKs) and DYRK2, 3, and 4 (class 2 DYRKs) (reviews: [12,13,14]). The CLK family comprises 4 members: CLK1, 2, 3, and 4 (review: [15]).

Alignment of DYRKs and CLKs sequences shows the classical central kinase catalytic domain flanked by N-terminal and C-terminal extensions (Figure 3 and Figure 4). The N-terminal domain of all DYRKs displays a conserved DYRK homology box (DH) [16] that contributes to autophosphorylation of a conserved tyrosine in the kinase domain (Tyr321 in DYRK1A) during maturation of the kinase [17,18]. Autophosphorylation on the tyrosine residue is preceded by hydroxylation of a proline residue by the PHD1 prolyl hydroxylase, an absolute requirement for catalytic activation of the kinase [19]. The N-terminal domain of all DYRKs except DYRK3 contains a nuclear localization signal domain (NLS) [20]. DYRK2, DYRK3, and DYRK4 contain a conserved N-terminal autophosphorylation accessory (NAPA) domain essential for autophosphorylation of the activation loop tyrosine [21]. The C-terminal domain of DYRK1A and DYRK1B displays a region enriched in proline, glutamic acid, serine, and threonine known as a PEST sequence, which favors rapid degradation [22]. A region containing 13 consecutive histidine residues is present in the C-terminal region of DYRK1A but not in other DYRKs or CLKs. A comprehensive analysis of the human proteome revealed that only 86 proteins display such a histidine repeat stretch (5 or more histidines) [23]. The presence of a homopolymeric histidine repeat in nuclear proteins appears to be involved in the targeting/localization of these proteins to the nuclear speckles compartment. Many of these polyhistidine sequence-bearing proteins are expressed in the nervous system [23]. The unique polyhistidine sequence provides a natural His-tag which allows the purification/enrichment of DYRK1A using immobilized metal-affinity chromatography (IMAC) (nickel, cobalt) [24,25] [Sévère et al., unpublished]. DYRKs and CLKs have been highly conserved throughout evolution, and orthologs are found in yeast [26,27], plants [28,29,30,31,32], unicellular algae [33,34], and unicellular parasites such as *Trypanosoma* [35,36,37], *Leishmania* [38,39,40], and *Plasmodium* [41,42,43,44,45,46].

Crystal structures of various DYRKs and CLKs, alone or in complex with inhibitors, have been solved (Table 2). These structures have allowed a detailed understanding of the mechanism of activation of DYRKs by autophosporylation on the tyrosine residue as well as an understanding of the binding mode of numerous inhibitors, providing very useful information for the structure-guided synthesis of improved pharmacological inhibitors.

**Table 2:** Crystal structures of DYRKs and CLKs alone or in complex with inhibitors.

Kinase	Ligand	PDB	Reference
**DYRK1A**	DJM2005	2VX3, 2WO6	[18]
	Leucettine L41	4AZE	[43]
	Harmine	3ANR	[44]
	INDY	3ANQ	[44]
	Compounds 3 and 23	4MQ1, 4MQ2	[45]
	LDN-211898	5AIK	Elkins, unpublished
	PKC412	4NCT	[46]
	Inhibitor 5t, 5s	4YLL, 4YLK	[47]
	Compound 32, 14	6A1G, 6A1F	[48]
	XMD7-112, JWD-065	6EJ4, 6EIV	[49]
	[b]-annulated chloro-substituted indole 13	4YLJ	[50]
	KuFal319	6T6A	[50]
	AnnH75	4YU2	[51]
	compound 2-2 (harmine derivative)	6UWY	[52]
	GNF2133	6UIP	[53]
	DJM2005 (DB07608)	2WO6	[18]
**DYRK2**	-	3K2L	[18]
	Leucettine L41	4AZF	[43]
	Indirubin 6i	3KVW	[54]
	EHT 5372, EHT 1610	5LXC, 5LXD	[55]
**DYRK3**	Harmine	5Y86	[56]
**CLK1**	-	6TW2	[57]
	compounds 8g, 16	6FT8, 6FT9	[58]
	debromohymenialdisine	1Z57	[59]
	KH-CB19	2VAG	[60,61]
	Pyrido [3, 4-G] quinazolines 13, 14	5J1V, 5J1W	[62]
	Compound 25	5X8I	[63]
	CX-4945	6KHD	[64]
	CX-4945	6FYO	[65]
	Compounds 9m, 10i	6Q8P, 6Q8K	[66]
	5-iodotubercidin	6G33	[67]
	furanopyrimidines VN412, VN316, VN345	6I5H, 6I5L, 6I5K	[68]
	ETH1610 (Cpd 17)	6YTI	[69]
	KH-CARB13 (Cpd 3)	6YTG	[69]
	Tg003 (Cpd 2)	6YTE	[69]
	GW807982X (Cpd 8)	6ZLN	[69]
	imidazopyridazine (Cpd 1)	6YTA	[69]
	CAF052	7AK3	[70]
**TbCLK1**	AB1	6Q2A	[40]
**CLK2**	1RO, NR9	3NR9	Knapp, unpublished
	CX-4945	6KHE	[64]
	CX-4945	6FYL	[65]
**CLK3**	-	2EU9, 2EXE	[59]
	KH-CB19	2WU7	[60]
	K00546	2WU6	[60]
	Leucettine L41	3RAW	[71]
	CX-4945	6KHF	[64]
	CX-4945	6FYP	[65]
	KH-CARB13 (Cpd 3)	6YU1	[69]
	Tg003	6YTW	[70]
	compound 8a	6FT7	[58]
**CLK4**	CX-4945	6FYV	[65]

The nuclear interactome of DYRK1A is highly enriched in DNA damage repair factors (RNF169), transcriptional elongation factors, and E3 ubiquitin ligases [72,73,74]. The interactome of all CMGC kinases, including DYRKs and CLKs, has been extensively studied [75]. Other large-scale interactome studies provide information on proteins binding to DYRKs and CLKs [76,77]. A detailed description of the DYRKs and CLKs interactomes is beyond the scope of this review. However, we would like to mention WDR68, also known as DCAF7 (DDB1-associated and CUL4-associated factor 7) or HAN11 (Human homolog of the *Petunia hybrida an11* gene), a scaffolding protein of the WD40-repeat protein family [78] that binds class 1 DYRKs and HIPK2 (Homeodomain-interacting protein kinase 2). The interaction between WDR68 and DYRK1A/DYRK1B has been extensively studied [79,80,81]: it involves a conserved 12 amino acid sequence located in the N-terminal domain of DYRK1A/1B. This interaction mediates binding to other proteins, such as the adenovirus E1A oncoprotein [81] and RNA polymerase II [82], thereby probably favoring substrate recruitment for DYRK1A/1B and HIPK2. WDR68 is essential for craniofacial development, a process involving DYRK1A [83,84]. DYRK1A regulates the interaction between WDR68 and Huntington-associated protein 1 (Hap1), which may contribute to postnatal growth retardation in Down syndrome (DS) [85]. Expression of WDR68 regulates the level of expression of DYRK1A and DYRK1B [86].

DYRK and CLK kinases phosphorylate many substrates involved in signaling pathways, mRNA splicing, chromatin transcription, DNA damage repair, cell survival, cell cycle control, differentiation, homocysteine/methionine/folate regulation, endocytosis, neuronal development and functions, synaptic plasticity, etc. Reviewing substrates and cellular functions of all DYRKs and CLKs is beyond the scope of this brief review, although phosphorylation of substrates and their cellular and physiological consequences underlie normal functioning and pathological conditions.

## 2. DYRKs and Human Disease

**Figure 5:**
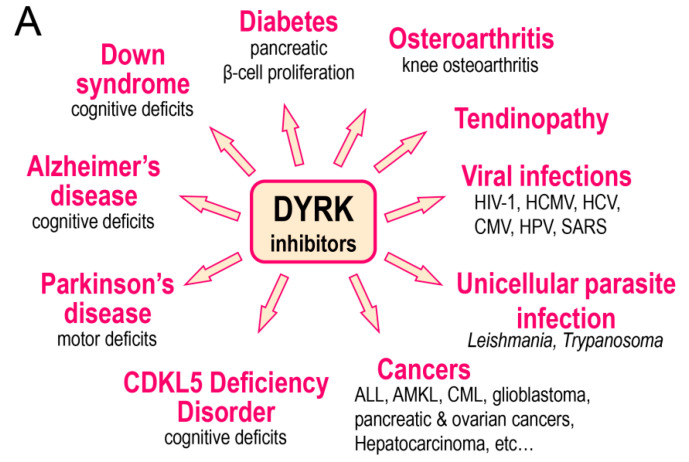

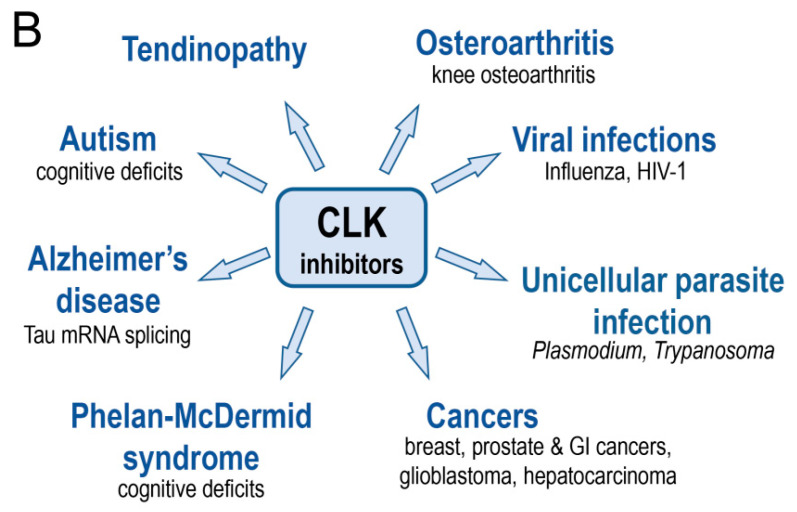
DYRK and CLK inhibitors and their potential use. (**A**) DYRK inhibitors (in particular inhibitors of DYRK1A) have been investigated in the indicated diseases. (**B**) CLK inhibitors (in particular inhibitors of CLK1) have been investigated in the indicated diseases.

There is growing evidence for the involvement of various DYRKs in human disease. We will briefly review these accumulating data (Table 3 and Figure 5A).

### 2.1. DYRK1A and Down Syndrome (DS)

The gene encoding DYRK1A is located on chromosome 21, within the Down syndrome critical region (DSCR), the triploidy of which is responsible for most DS-associated deficiencies (reviews: [13,14]) (Table 3 for more details). There is considerable genetical and pharmacological evidence showing that the mere 1.5-fold overexpression of DYRK1A is responsible for most cognitive deficits observed in DS patients (reviews: [14,87,88,89,90,91,92]). Genetical normalization of DYRK1A levels or pharmacological inhibition of its catalytic activity restores cognitive functions (Table 3 for specific references). The development of pharmacological inhibitors of DYRK1A is a major avenue for the treatment of cognitive deficits associated with DS (and Alzheimer’s disease) (reviews: [88,89,93]).

### 2.2. DYRK1A and Alzheimer’s Disease (AD)

There is mounting evidence for a role of DYRK1A in the onset of AD (reviews: [14,88,94,95]) (Table 3 for more details). DYRK1A phosphorylates key substrates involved in AD and dementia: Tau, septin 4, amyloid precursor protein (APP), presenilin 1, neprilysin, Munc18-1, α-synuclein, RCAN1, and β-tubulin. By modulating alternative splicing of Tau exon 10, DYRK1A favors the production of the 3R-Tau splice isoform (characteristic for DS/AD/tauopathy) over the 4R-Tau isoform [96,97,98]. Inhibition of DYRK1A and possibly of other DYRKs and CLKs promotes autophagy, which could counterbalance the autophagy deficit seen in AD.

### 2.3. DYRK1A and Parkinson’s Disease (PD)

Genome-wide association studies (GWAS) have revealed that DYRK1A is a risk factor for PD [99]. DYRK1A phosphorylates key factors for PD such as parkin, septin 4, and α-synuclein. Upregulation of micro-RNAs specific for PD targets DYRK1A expression [100]. There is further evidence that DYRK1A expression is increased in PD and in Pick’s disease [101].

### 2.4. DYRK1A and Mental Retardation Disease 7 (MRD7)

Haploinsufficiency of the DYRK1A gene, due to various truncation mutations, microdeletions, or missense variants resulting in reduced DYRK1A, is responsible for MRD7, an autism spectrum disorder displaying microcephaly, intellectual disability, speech impairment, and distinct facies (reviews: [91,102,103,104]).

### 2.5. DYRK1A and Viral Infections

DYRK1A and DYRK1B are utilized during human cytomegalovirus (HCMV) placental replication. Inhibition of DYRKs prevent replication of various viruses, including hepatitis C virus (HCV), human cytomegalovirus (HCMV), human immunodeficiency virus type 1 (HIV-1), and herpes simplex virus 1 (HSV-1) (Table 3 for more details).

### 2.6. DYRK1A and Diabetes

There is a growing body of evidence showing that DYRK1A/1B inhibitors induce the proliferation of insulin-producing pancreatic β-cells, making DYRK1A/1B kinases attractive therapeutic targets for β cell regeneration for both type 1 and type 2 diabetes [105,106] (Table 3 for more details).

### 2.7. DYRK1A and Cancers and Leukemias

There is abundant literature linking DYRK1A with solid cancers and leukemias (reviews: [107,108,109]). The most prominent examples are pancreatic cancer, brain tumor, acute megakaryoblastic leukemia (AMKL) [110], and acute lymphoblastic leukemia (ALL) [111] (Table 3 for more details). DYRK1A regulates DNA damage response [72,74]. In some situations, DYRK1A appears to function as a tumor-suppressor protein [112,113,114].

### 2.8. Other DYRKs and Human Disease

DYRK1B is involved in the replication of various viruses including HCV, Chikungunya virus, Dengue virus, SARS coronavirus, HCMV, and human papillomavirus (HPV). Like with DYRK1A, DYRK1B inhibition leads to the proliferation of pancreatic, insulin-producing β-cells. DYRK1B is involved in neuroinflammation [115]. Targeting DYRK1B provides a new rationale for treatment of various solid cancers such as liposarcoma or breast cancers (reviews: [116,117]) as well as in chronic myeloid leukemia (CML).

DYRK2, in association with GSK-3β, regulates neuronal morphogenesis [118]. DYRK2 is involved in various ways in cancer development (reviews: [119,120]).

DYRK3 promotes hepatocellular carcinoma [121] and glioblastoma [122]. DYRK3 is required for influenza virus replication [123]. DYRK3 couples stress granule condensation/dissolution to mechanistic target of rapamycin complex 1 (mTORC1) signaling [124]. DYRK3 regulates phase transition of membraneless organelles in mitosis [125]. DYRK3 and DYRK4 are involved in the regulation of cytoskeletal organization and process outgrowth in neurons.

DYRK1A decreases axon growth, DYRK3 and DYRK4 increase dendritic branching, and DYRK2 decreases both axon and dendrite growth and branching [126].

**Table 3:** DYRKs and human disease. Evidence for causality and beneficial effects of pharmacological treatment by DYRKs inhibitors.

Kinase Target	Disease	References
DYRK1A	Down syndrome (DS)	[127,128,129,130,131,132,133,134,135,136,137,138,139,140,141,142,143,144,145,146,147]
DYRK1A	Alzheimer’s disease (AD) and other Taupathies	[96,98,128,129,131,148,149,150,151,152,153,154,155,156,157,158,159,160,161,162,163]
DYRK1A	Parkinson’s disease	[99,100,101,131,164,165,166,167,168]
DYRK1A	Pick’s disease	[101]
DYRK1A	CDKL5 Deficiency Disorder	[169]
DYRK1A	Diabetes	[52,53,105,106,170,171,172,173,174,175,176,177,178,179]
DYRK1A	Regulation of folate and methionine metabolism	[180]
DYRK1A	Cancers (review)	[109]
DYRK1A	Glioblastoma	[181]
DYRK1A	Head and neck squamous cell carcinoma	[182]
DYRK1A	Pancreatic ductal adenocarcinoma	[183,184,185]
DYRK1A	Hepatocellular carcinoma	[186]
DYRK1A	Ovarian cancer	[187,188]
DYRK1A	Acute megakaryoblastic leukemia (AMKL)	[110,189]
DYRK1A	Acute lymphoblastic leukemia (ALL)	[111,190,191]
DYRK1A	Psoriasis	[192]
DYRK1A	Knee osteoarthritis	[193,194]
DYRK1A	Tendinopathy	[195]
DYRK1A	Human immunodeficiency virus type 1 (HIV-1)	[196,197,198]
DYRK1A DYRK1B	Human cytomegalovirus (HCMV)	[199]
DYRK1B	Hepatitis C virus (HCV), Chikungunya virus, Dengue virus, and severe acute respiratory syndrome (SARS) coronavirus Cytomegalovirus (CMV) Human papillomavirus (HPV)	[199,200,201]
DYRK1B	Diabetes	[105]
DYRK1B	Neuroinflammation	[115]
DYRK1B	Oral squamous cell carcinoma Liposarcoma Breast cancer Hedgehog/GLI-dependent cancer	[117,202,203,204,205]
DYRK2	Cancers (reviews)	[119,120,206,207]
DYRK2	Triple-negative breast cancer (TNBC) and multiple myeloma (MM)	[208,209]
DYRK2	Lung adenocarcinoma	[210]
DYRK2	Chronic myeloid leukemia (CML)	[211,212]
DYRK2	Gliblastoma	[213]
DYRK2	Colorectal cancer (tumor suppressor)	[214]
DYRK2	Liver cancer (predictive marker)	[215]
DYRK2	*Trypanosoma cruzi*	[216]
DYRK3	Hepatocellular carcinoma	[121]
DYRK3	Glioblastoma	[122]
DYRK3	Influenza virus replication	[123]
DYRK3	Anemia	[217]
DYRK3	Osteoarthritis	[218]
DYRK4	Breast cancer	[219]
DYRKs	Glioblastoma	[220]
DYRKs	Herpes virus, rhesus macaque cytomegalovirus, varicella-zoster virus, and herpes simplex virus (HSV-1)	[221]
LmDYRK1	Leishmaniasis	[39]
TbDYRK	*Trypanosoma brucei* (sleeping sickness)	[35,36,37]
DYRKs/CLKs	Glioblastoma	[220]

## 3. CLKs and Human Disease

The data supporting the involvement of various CLKs in human disease is briefly described below and in Table 4 and Figure 5B.

CLKs play essential functions in alternative splicing. CLKs act as a body-temperature sensors, which globally control alternative splicing and gene expression. The activity of CLKs is indeed highly responsive to physiological temperature changes, which is conferred by structural rearrangements within the kinase activation segment [57].

CLK1 triggers periodic alternative splicing during the cell division cycle [222]. CLK1 regulates influenza A virus mRNA splicing, and its inhibition prevents viral replication. CLK1 and CLK2 also regulate HIV-1 gene expression. CLK1 is an autophagy inducer. CLK1 inhibition may prevent chemoresistance in glioma, and CLK1 inhibition by TG693 allows the skipping of mutated exon 31 of the dystrophin gene in Duchenne Muscular Dystrophy. CLK1 autoregulates itself through exon skipping and intron retention [223].

Inhibition of CLK2 has been proposed as a way to improve neuronal functions and combat intellectual disability and autism in Phelan–McDermid syndrome (PMDS) [65]. Alternative splicing of Tau exon 10 is regulated by CLK2 and other CLKs, leading to changes in the 3R/4R isoform ratio and neurodegeneration in sporadic AD [224,225]. Dual inhibitions of CLK2 and DYRK1A by Lorecivivint (SM04690) and by its analogue SM04755 are potential disease-modifying approaches for knee osteoarthritis [193,194] and for tendinopathy, respectively [195]. CLK2 inhibition compromises MYC-driven breast tumors, triple-negative breast cancer, and glioblastoma. Inhibition of CLK2, CLK3, and/or CLK4 blocks HIV-1 production.

CLK3 contributes to hepatocellular carcinoma [226], prostate cancer [227], and cholangiocarcinoma [228]. CLK3 is abundantly expressed in testis and in spermatozoa.

**Table 4:** CLKs and human disease. Evidence for causality and beneficial effects of pharmacological treatment by CLK inhibitors.

Kinase Target	Disease	References
CLK1	Glioblastoma Small-cell lung cancer	[229] [230]
CLK1	Duchenne muscular dystrophy	[231]
CLK1	Influenza A West Nile and Chikungunya viruses	[232,233,234,235,236] [61]
CLK1/CLK2	Triple-negative breast cancer	[237]
CLK2	HIV-1	[238]
CLK2	Autism Phelan-McDermid syndrome (PMDS)	[239] [65]
CLK2	Knee osteoarthritis Tendinopathy	[193,194] [195]
CLK2	Breast cancer Triple-negative breast cancer Glioblastoma	[240,241] [242,243] [244,245]
CLK2	Alzheimer’s disease (alternative splicing of Tau exon 10)	[224,225]
CLK3	Hepatocellular carcinoma Prostate cancer Cholangiocarcinoma	[226] [227] [228]
CLKs	Body temperature	[57]
CLKs	Prostate cancer Gastrointestinal cancer Colorectal, ovarian cancers	[227] [246] [247]
PfCLKs	*Plasmodium falciparum* (malaria)	[248,249,250,251,252,253]
Tb CLK1/2	*Trypanosoma brucei* (sleeping sickness)	[38,40]

## 4. Therapeutic Potential of DYRK and CLK Inhibitors

**Figure 6:**
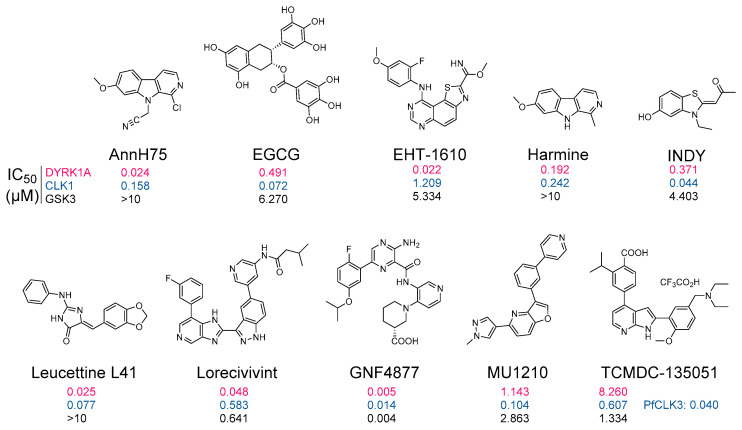
DYRK and CLK inhibitors. A few representative pharmacological inhibitors: AnnH75 [51], EGCG [255], EHT-1610 [55], Harmine [256], INDY [44], Leucettine L41 [43,257], Lorecivivint [258,259], GNF4877 [170,179], MU1210 [68], and TCMDC-135051 [251,252]. Numbers under each structure indicates IC50 values (expressed in µM) towards DYRK1A, CLK1, and GSK3β (33PanQinase™ assay, Reaction Biology Corp.).

Abnormal activities in DYRKs and CLKs have motivated numerous groups to search for, optimize, and characterize pharmacological inhibitors of these kinases for their use in various indications (reviews: [88,89,93]) (Figure 5). There is particular interest in the development of DYRKs/CLKs inhibitors as potential drug candidates to address cognitive deficits in DS and AD as well as to increase the pancreatic β-cell mass in both type 1 and type 2 diabetes (review: [106]) or to inhibit several cancers and leukemias by inhibiting cell proliferation. A few representative inhibitors are shown in Figure 6. Most DYRK1A inhibitors also inhibit, to various extent, DYRK1B, 2, 3, and 4 as well as the closely related CLK1, 2, 3, and 4 [93]. Apart from FINDY, which inhibits DYRK1A by interfering with its folding process [254], all reported inhibitors appear to act by competing with ATP in its binding to the catalytic site of the kinases (as demonstrated by enzymological studies as well as by co-crystallization with their kinase targets (Table 2)). Several DYRK1A inhibitors have been reported in recent years (reviews: [88,93,95]) which, like Leucettines and Leucettine L41 in particular, correct cognition deficits in DS and AD animal models [127,128,148].

## 5. Conclusions

The limited studies that have been carried out so far with DYRKs and CLKs have opened up new avenues in our understanding of their regulation and functions. Yet, a great deal of work remains to be done to fully understand the cellular and physiological functions of each member of the DYRK and CLK families. Tissular and cellular distribution, polymorphism and mutations, regulation of expression levels, and post-translational modifications are just a few of the parameters that need to be investigated in detail. Conditional knock-out/knock-in and overexpression models will also contribute to the understanding of the unique roles of each of these kinases and their eventual redundancy. Very precious tools—antibodies, affinity reagents, pharmacological inhibitors, kinase inactive mutants, transgenic animals—have been developed, yet DYRK1A has been mostly studied, and other DYRKs and CLKs will require the development of specific tools.

The currently available data demonstrate major implications of several protein kinases of the DYRK and CLK families in several human diseases. The first inhibitors are reaching regulatory preclinical studies and early clinical studies. The next few years will certainly see the validation of specific DYRKs and CLKs inhibitors for specific clinical indications. It is still a bit early to speculate which one these will be. Clearly though, cognition in DS and AD, diabetes, cancers, and osteoarthritis are the most advanced examples of potential applications, but viral and unicellular parasite infections will certainly gain momentum as potential therapeutic indications for DYRKs/CLKs inhibitors. Higher potency and higher selectivity will also emerge in the near future. We can clearly anticipate that, as fundamental knowledge will accumulate on these protein kinases, more applied pharmaceutical work will result in well characterized, selective, and potent inhibitors leading to significant clinical improvements for patients.

## Figures and Tables

**Table 1:** Sequence comparison of human CLK and DYRK family members. Numbers indicate percentage sequence identity and similarity among the nine kinase domains. Sequences were obtained from UniProtKB, and % of similarity and identity were calculated using BlastP (https://blast-ncbi-nlm-nih-gov.cov) (accessed on 1 June 2021).

	%Identify	CLK				DYRK				
%Similarity		1	2	3	4	1A	1B	2	3	4
CLK	1	100	67	62	87	30	33	36	36	33
	2	84	100	73	68	32	31	32	30	32
	3	77	87	100	64	31	31	33	33	35
	4	93	84	79	100	30	31	34	35	32
DYRK	1A	48	51	50	48	100	85	45	43	45
	1B	49	49	48	48	93	100	45	44	45
	2	55	52	53	53	60	61	100	79	59
	3	55	51	52	54	63	64	89	100	57
	4	54	56	55	55	62	63	74	72	100

## Abbreviations

AD	Alzheimer’s disease
AGC	PKA, PKG, and PKC family
ALL	acute lymphoblastic leukemia
AMKL	acute megakaryoblastic leukemia
APP	amyloid precursor protein
CAMK	Ca^2+^/calmodulin-dependent kinases
CDKs	cyclin-dependent kinases
CSNK1/CK1	casein kinases 1
CK2	casein kinase 2
CLKs	cdc2-like kinases
CML	chronic myeloid leukemia
DCAF7	DDB1-associated and CUL4-associated factor 7
DH	DYRK homology box
DS	Down syndrome
DYRKs	dual specificity, tyrosine phosphorylation regulated kinases
GSK3	glycogen synthase kinase 3
GWAS	genome-wide association studies
HAN11	human homolog of the Petunia hybrida an11 gene
Hap1	Huntington-associated protein 1
HCMV	human cytomegalovirus
HCV	hepatitis C virus
HIV-1	human immunodeficiency virus type 1
HIPK2	Homeodomain-interacting protein kinase 2
HPV	human papillomavirus
HSV-1	herpes simplex virus 1
IMAC	immobilized metal-affinity chromatography
MAPKs	mitogen-activated protein kinases
MRD7	mental retardation disease 7
NAPA	N-terminal autophosphorylation accessory domain
NLS	nuclear localization signals domain
PD	Parkinson’s disease
PEST	region enriched in proline (P), glutamic acid (E), serine (S), and threonine (T) residues
PKA	cAMP-dependent protein kinase
PKC	protein kinase C
PKG	cGMP-dependent protein kinase
PMDS	Phelan-McDermid syndrome
STE	homologs of yeast STE7, STE11, and STE20 kinases
TK	tyrosine kinases
TKL	tyrosine kinase-like kinases
WDR68	WD40-repeat protein 68
